# Yeast Stress Response to Synthetic Constructs

**DOI:** 10.1021/acssynbio.5c00715

**Published:** 2026-01-30

**Authors:** Musa Tartik

**Affiliations:** † Department of Molecular Biology and Genetics, Faculty of Arts and Sciences, 162312 Bingol University, Bingol 12000, Turkey; ‡ Department of Biochemistry & Biophysics, The University of North Carolina, Chapel Hill, North Carolina 27599, United States

**Keywords:** synthetic biology, yeast stress response, stressome, *Saccharomyces cerevisiae*, metabolic burden

## Abstract

*Saccharomyces cerevisiae* is widely adopted as a chassis in synthetic biology. However, heterologous constructs often disrupt proteostasis, metabolism, redox balance, and secretory processes. These disruptions activate a complex network of stress pathways. These include the heat shock response, unfolded protein response, oxidative stress defenses, cell wall integrity signaling, the high-osmolarity glycerol pathway, and Snf1/AMPK-mediated energy regulation. Collectively, these pathways form a stressome that maintains cellular homeostasis but constrains productive capacity. A comprehensive understanding of how synthetic designs interact with these pathways is essential for developing robust yeast systems. Strategies such as promoter tuning, chaperone augmentation, redox and cofactor balancing, lipid and membrane optimization, dynamic regulation, and pathway compartmentalization can reduce cellular burden. Emerging methods also improve stress mitigation. These include CRISPR-based circuit rewiring, adaptive laboratory evolution, synthetic organelle construction, and data-driven strain engineering. This review summarizes construct-induced stress in engineered yeast and presents stress-aware design principles to advance more resilient, higher-yielding *S. cerevisiae* strains for biotechnology.

## Graphical abstract

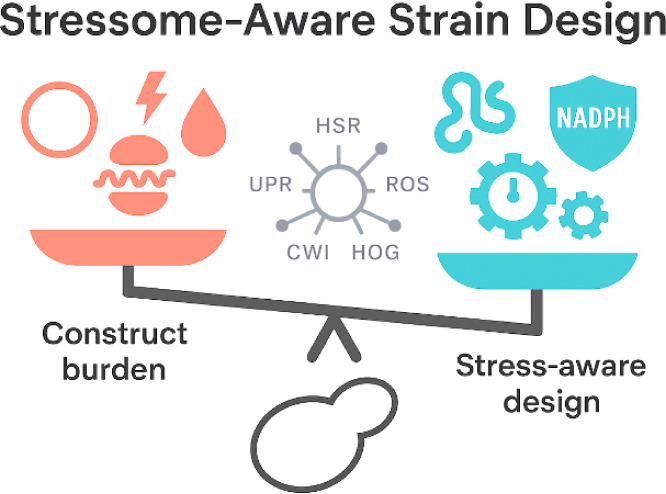

## 1 Introduction

*Saccharomyces cerevisiae* is pivotal in industrial biotechnology for its genetic tractability, robust metabolism, and use in producing fuels, chemicals, and bioproducts. Its efficient homologous recombination and accessible genome enable rapid assembly of synthetic pathways, modules, and precise regulatory networks.^1–6^ As synthetic designs grow more complex and expression burdens increase, engineered strains face stresses that limit growth, stability, and yield. Understanding interactions between synthetic constructs and stress pathways is essential for developing high-performing yeast strains.

Synthetic constructs introduce proteostatic, metabolic, redox, and secretory stress. High-copy plasmids and strong promoters increase transcriptional and translational demands, leading to misfolded protein accumulation and activation of the heat shock response (HSR) via Hsf1-dependent pathways.^7–11^ Misfolded proteins cause dosage-dependent fitness penalties and induce unfolded-protein responses.^11^ Producing heterologous secretory proteins often overwhelms the endoplasmic reticulum (ER), triggering the unfolded protein response (UPR) via the Ire1–Hac1 pathway.^12–14^ High-flux metabolic pathways disrupt redox homeostasis, generate reactive oxygen species (ROS), and activate Yap1 and Skn7.^15–18^ These stress response pathways protect cells but divert ATP, amino acids, and chaperone resources from biosynthetic processes, creating a trade-off between stress tolerance and productivity.

To address this trade-off, recent research has focused on metrics that account for the energetic costs of cellular stress. One framework is the ATP-adjusted productivity index, which normalizes product yield to ATP costs for proteostasis, transcription, and protein turnover. This approach extends foundational studies on the energy demands of ribosomes and protein biosynthesis^8,9,19^ and recent estimates of protein production costs at the cellular level.^20^ Despite their promise, these burden-adjusted metrics need standardized assumptions and reporting. Such standards are essential for meaningful cross-study comparisons.

Stress marker panels offer practical ways to quantify construct burden using transcriptional reporters or biosensors responsive to pathways such as HSP26 or SSA1 (HSR), KAR2 or spliced HAC1 (UPR), and fluorogenic dyes (DHE, H_2_DCFDA) for ROS. Recent advances combine stress-responsive promoters with fluorescent reporters, enabling real-time measurement via flow cytometry or time-lapse microscopy.^7,12,14,15,21,22^ Alongside ATP assays, metabolomics, and growth metrics (μmax, biomass-specific productivity), these tools systematically evaluate construct burden for various designs and experimental conditions.

Systems-level investigations continue to clarify the regulatory topology of yeast stress responses. Hsf1 regulates the HSR by upregulating chaperones and proteostasis factors.^7,23^ The Ire1–Hac1 signaling cascade maintains ER homeostasis by activating the UPR, which responds to the accumulation of unfolded proteins.^12,13^ Transcription factors Yap1 and Skn7 coordinate the defense against oxidative stress, functioning as a protective mechanism against cellular damage.^15,16^ Elucidating these mechanisms supports rational strain engineering. For instance, coexpression of ER chaperones such as Kar2/BiP or Pdi1 can alleviate UPR burden, analogous to optimizing traffic flow to prevent congestion. This strategy improves secretion efficiency in recombinant production systems.^14,24–27^ Furthermore, engineering antioxidant pathways or modulating redox cofactor balance can stabilize strains subjected to oxidative stress during high-flux metabolic activity, similar to reinforcing a levee to prevent flooding^17,18,28^

Despite significant advances, important gaps remain. Determining how specific genetic constructs elicit distinct mechanistic stress responses and how these architectures quantitatively influence yeast burden tolerance limits remains challenging. This review consolidates current knowledge on construct-induced burden, synthesizes methodological approaches for stress quantification, and highlights engineering strategies such as promoter tuning, chaperone coexpression, and dynamic stress-responsive regulation to enhance strain robustness. By connecting molecular mechanisms to design principles, this article provides a framework for developing synthetic yeast strains that sustain high productivity within complex engineered pathways.

## 2 Mechanistic Foundations of Yeast Stress Responses

This section examines the main stress response pathways in *S. cerevisiae*. It emphasizes how these interconnected systems are activated by synthetic constructs. These pathways influence cellular outcomes. For each pathway, the underlying mechanisms, regulatory nodes, and cross-pathway interactions are analyzed. Current engineering strategies for stress mitigation and productivity enhancement are also discussed. A mechanistic perspective is critical for designing interventions in synthetic yeast strains.

### 2.1 The HSR: Integrating Cytosolic Proteostasis with ER and Redox Stress

The HSR is the main eukaryotic defense against proteotoxicity. It acts as a general stress module and responds to folding loads from multiple sources. In *S. cerevisiae*, the master transcription factor Hsf1 is usually kept inactive by transient interactions with Hsp70 and Hsp90. When nascent or misfolded peptides exceed the capacity of these chaperone pools, Hsf1 is released. This triggers upregulation of repair machinery, including the Ssa1–4 refolding complex, Hsp104 disaggregase, and ubiquitin-proteasome factors.^7,29–31^ The HSR was historically linked to thermal stress, but it is now central in synthetic biology. Heterologous expression often activates the HSR. This is not due to protein instability, but rather because rapid translation from strong promoters can exceed the availability of cytosolic chaperones. As a result, non-native intermediates may accumulate.^32–34^

The HSR operates in a tightly connected “tri-partite” regulatory network with the UPR and oxidative stress signaling. As a result, ER-originating stresses often reach the cytosol. For example, high-level secretion needs extensive oxidative folding from the Ero1p–Pdi1p relay. This process generates ROS, which diffuses into the cytosol and oxidizes Hsf1 or its repressors. As a result, the HSR is triggered even in the absence of cytosolic misfolding.^7,35^ When ER-associated degradation (ERAD) pathways are saturated, misfolded proteins may overflow from the ER. This causes cross-compartmental competition for chaperones.^36–38^ Strong Hsf1 activation has also been reported to reduce HAC1 mRNA splicing and dampen UPR signaling.^7^ This crosstalk causes Hsf1 activation to show nonlinear, threshold-like behavior. The system is mostly inactive until a critical level of stress is reached. After this, the response becomes rapid, sustained, and metabolically expensive.^30,39^

These insights show that strain design should go beyond simple overexpression. Chronic HSR activation is costly since Hsp70-mediated refolding uses ATP that would normally support biomass accumulation.^9^ Engineering should prioritize resource-aware promoter tuning. Expression strength should stay just below the nonlinear Hsf1 activation threshold. This prevents unnecessary use of translational resources.^8,9^ Constitutive chaperone overexpression can drain energy reserves. Instead, some recent approaches use stress-responsive promoters to decouple chaperone synthesis from the growth phase.^40^ By balancing cytosolic cochaperone levels with ER-specific folding factors and antioxidant defenses, engineered strains can handle high production without destabilizing the proteostasis network.

### 2.2 The UPR: A Comprehensive Analysis of ER Homeostasis

The primary challenge in high-level production of recombinant secretory proteins is the economic burden of misfolded protein accumulation. Misfolded proteins significantly disrupt cellular function and limit productivity. The UPR is a critical stress-response pathway. It is activated by the accumulation of misfolded proteins in the ER, a condition frequently encountered during high-level production of recombinant secretory proteins.^13,41,42^ In *S. cerevisiae*, the ER stress sensor Ire1 recognizes these unfolded proteins. Ire1 then oligomerizes and autophosphorylates to activate its cytoplasmic RNase domain.^43,44^ This activation leads to the unconventional splicing of a specific intron in the HAC1 mRNA. The spliced mRNA is then translated into the transcription factor Hac1p.^37,45^ Hac1p upregulates genes required for protein folding, such as KAR2 and PDI1. It also upregulates genes involved in ER expansion and ERAD. Collectively, these processes enhance the ER’s capacity for protein processing.^42^ In yeast, the Ire1–Hac1 pathway represents the sole highly conserved branch of the UPR. This contrasts with the multiple branches present in metazoans.^13,46,47^

Following the restoration of ER homeostasis, Ire1 undergoes dephosphorylation and dissociation. This event terminates HAC1 mRNA splicing and reduces the expression of Hac1p target genes.^43,48^ Prolonged UPR activation leads to ATP depletion due to increased synthesis of ER chaperones, ER expansion, and elevated ERAD activity.^48–50^ Higher resource consumption directly impacts cellular metabolism, growth, and productivity.^51,52^

UPR activation is closely associated with oxidative stress responses. Protein folding within the ER generates ROS.^53–56^ A prolonged UPR can trigger apoptosis-like pathways and significantly compromise cell survival in production environments.^43,57–59^ The UPR also interacts with other major stress response pathways. For instance, ER stress can activate the Cell Wall Integrity (CWI) pathway, the High-Osmolarity Glycerol (HOG) pathway, and the Snf1/AMPK pathway. All are linked to metabolic stress. These pathways can also reciprocally influence ER homeostasis.^56,60–63^

A key study investigated the yeast stress response to improve α-amylase secretion.^52^ High-level α-amylase expression was hypothesized to lead to the accumulation of misfolded proteins and to induce cellular stress. Researchers focused on three interventions to address this: splicing HAC1, overexpressing INO1, and overexpressing SSO2. Coexpression of the spliced, active HAC1 form resulted in sustained chaperone upregulation. Valkonen et al. reported a 70% increase in α-amylase secretion.^52^ Additional studies have shown that overexpressing INO1 enhances membrane biosynthesis.^64,65^ Overexpressing SSO2 improves vesicle trafficking.^42,66^ Although these strategies are often discussed collectively, no single study has combined all three or quantified their combined effects. Nevertheless, the underlying mechanisms are well established. Simultaneously addressing these bottlenecks reduces UPR activation and increases protein production. Future studies should systematically combine and quantify these interventions to better understand their synergistic effects. This approach will guide research priorities in the field and provide a clearer path for optimizing yeast strains for industrial applications.

To practically implement these findings, practitioners could adopt a stepwise experimental approach in their laboratories. Initially, they can replicate the separate interventions of HAC1 form expression, INO1 overexpression, and SSO2 overexpression. Use control conditions for comparison. After establishing the individual effects, a combinatorial approach is recommended. Implement all three interventions concurrently to evaluate their synergistic potential. Such experiments can begin by setting standardized metrics for UPR activation and α-amylase secretion. Researchers should also monitor key stress markers and cellular growth rates. This systematic approach provides a practical starting point for optimization. It may also offer insights into additional genetic modifications that could enhance productivity and strain resilience in industrial applications.

A key insight for metabolic engineering is that ER folding capacity constrains secretory production. Modulating the UPR, such as by chaperone coexpression and enhanced vesicle trafficking, can improve secretion efficiency.^14,51,66^ However, these interventions require precise calibration to prevent chronic ER stress, which may impair cell growth and viability.^48,50,57^ To balance productivity gains with stress burden, researchers should aim for a UPR marker threshold that maximizes protein yield without inducing detrimental stress responses. For example, targeting a specific increase in Kar2p levels could serve as a guide for optimizing expression levels. The UPR functions not only as a response to misfolded proteins but also as a comprehensive ER remodeling and quality control program essential for cellular function.^13,41,45^

### 2.3 The Oxidative Stress Response: Precision Redox Balancing

Oxidative stress comes from excess ROS. This often follows increased metabolic flux or cofactor imbalances in engineered microbial strains.^7,15,67^ Excess flux depletes essential cofactors, such as NADPH, disrupts redox homeostasis, and increases ROS levels. ROS include hydrogen peroxide (H_2_O_2_), superoxide (O_2_^–^), and hydroxyl radicals (OH•), all generated as oxygen metabolism intermediates.^68^ In yeast, ROS mainly arise from mitochondrial respiration, peroxisomal oxidation, and cytochrome P450 activity.^69^ The transcription factors Yap1 and Skn7 are primary sensors. They induce antioxidant defenses such as catalases (CTA1), peroxidases (GPX3), and superoxide dismutases (SOD1, SOD2).^15,67,70^ Engineered pathways that use reducing cofactors, particularly NADPH, or produce redox-imbalanced metabolites, intensify ROS. This often leads to growth defects and cell damage.^71^

Yap1 and Skn7 also activate the thioredoxin and thioredoxin reductase pathways. These are central to the redox stress response.^7,15,72^ Yap1 detects H_2_O_2_ by oxidizing cysteine residues. This causes conformational changes that lead to nuclear localization and transcriptional activation.^73^ Oxidative stress also triggers DNA and protein repair, and defenses against membrane lipid peroxidation.^67,74^ However, measuring when these responses activate across different constructs and environments remains difficult.^15^ The challenge comes from limited instrument sensitivity, strain variability, and complex culture conditions.

Isobutanol biosynthesis imposes redox pressure on hosts. This occurs mainly due to poor NADPH regeneration and elevated ROS levels.^75,76^ Since isobutanol production relies on NADPH, a limited supply of this cofactor disrupts the redox balance. This raises ROS, lowers product yields, and affects cell survival.^77,78^ Boosting respiratory activity can increase flux toward isobutanol. But this also raises ROS, impairs protein synthesis, and slows growth.^79^

Redox-engineering often targets central carbon metabolism to improve NADPH regeneration.^80–84^ Overexpressing pentose phosphate pathway (PPP) enzymes such as ZWF1 and GND1 is common. This increases cellular NADPH and relieves cofactor bottlenecks.^85^ Adding exogenous electron mediators can enhance electron transfer and the use of redox cofactors. This also boosts the production of reduced metabolites, such as butanol.^78^ Stronger antioxidant defenses, such as overexpression of SOD2 and CTA1, reduce ROS and protect cells from oxidative stress.^75,83^ Combining these methods improves isobutanol yields and cell survival in yeast.^75^ Some constructs, such as certain β-glucosidase systems, have avoided a notable metabolic burden.^82^

In addition to the redox response, oxidative stress triggers broader networks. These include the HSR from proteotoxicity and the CWI pathway from membrane damage.^7,15,86^ Better antioxidant capacity usually protects against many stressors. Strengthening defenses helps against oxidative stress, heat shock, and cell wall damage. So, redox engineering can offer multiple benefits and improve metabolic engineering outcomes. Adding more NADPH supply or optimizing antioxidant expression is vital. These actions keep cells robust, reduce ROS-associated failures, and support stable, high-yield production.^15,67,84^ For example, such strategies can increase isobutanol yield in wild-type strains. These results highlight real advantages in engineered microbial systems and link models to applications.

### 2.4 The CWI Pathway: Dynamic Membrane and Wall Adaptation

The CWI pathway is a mitogen-activated protein kinase (MAPK) cascade. It is activated by membrane stress, including overproduction of membrane proteins or altered lipid profiles.^67,86^ Cell-surface sensors such as Wsc1–3 and Mid2 detect mechanical or structural changes in the cell wall.^87^ Upon stress detection, these sensors initiate the Rho1–Pkc1–MAPK signaling cascade. This leads to phosphorylation of the Slt2/Mpk1 MAP kinase. Phosphorylated Slt2/Mpk1 then induces transcription of genes responsible for cell wall biosynthesis, including chitin and glucan synthases. It also activates enzymes that remodel and reinforce the wall, thereby preserving cell structure and osmolarity.^88,89^

Synthetic biological constructs induce membrane stress through multiple mechanisms. These can be categorized as “design-induced” and “process-induced” stresses. Design-induced stress arises from the incorporation of heterologous membrane proteins. This compromises membrane integrity and stability, often seen as an increased proportion of unfolded membrane proteins. In contrast, process-induced stress arises from alterations in lipid biosynthesis due to metabolic burden and toxic metabolic byproducts.^90–92^ Ergosterol depletion indicates this state. Prolonged CWI pathway activation reallocates cellular resources toward wall repair. This leads to reduced growth rates and diminished long-term cell viability.^86,93^ The trade-off between cellular maintenance and metabolite production presents a key challenge in strain engineering. Strategic decisions are needed to optimize resource allocation for both cell integrity and productivity.

Metabolic engineering strategies aim to alleviate stress caused by the overexpression of foreign membrane proteins. Such proteins disrupt plasma membrane stability and activate stress pathways, such as the CWI cascade.^94,95^ These disruptions are typically associated with decreased growth and yield in yeast hosts.^95^ Altering membrane lipid composition is one approach to mitigate these effects. For instance, increasing unsaturated fatty acids by upregulating Δ9 desaturase OLE1 enhances membrane fluidity and stress tolerance.^96^ OLE1 overexpression confers increased resistance and activates related signaling pathways.^97^ Introducing additional desaturase activities further improves tolerance in engineered yeast.^98^ Such modifications also enhance resistance to freezing and salt stress.^99^ Targeting sterol biosynthesis, for example, by perturbing ERG6, alters membrane composition and influences growth, stress response, and drug resistance.^100,101^ Therefore, upregulation of OLE1 and other desaturases modulates sterol pathway activity and lipid profiles. These changes reduce chronic CWI activation and improve the performance of engineered yeast strains.

The CWI pathway interacts with the HOG pathway and the oxidative stress response. Together, these form a network of stress-adaptation mechanisms at the cell envelope.^67,102–104^ Lipid peroxidation from oxidative stress can activate the CWI pathway.^102,105^ In metabolic engineering, it is essential to realize that expressing membrane-bound proteins can trigger CWI activation via cell envelope stress. Proactive strategies, such as modifying lipid metabolism or selecting stress-tolerant protein variants, can mitigate this effect. Enhancing membrane fluidity and composition offers a direct way to counteract early stress signals. This approach helps maintain cell viability.^94,97,101^

### 2.5 The HOG Pathway: Directing Osmoadaptive Responses

The HOG pathway serves as the primary signaling module that enables *S. cerevisiae* to maintain osmotic homeostasis.^106,107^ Upon abrupt increases in external osmolarity, the MAP kinase Hog1 is activated. This results in a rapid accumulation of intracellular glycerol, which is essential for restoring turgor pressure. The increase in glycerol is accompanied by the transcriptional induction of osmoprotective genes that promote cell survival. Notably, synthetic constructs that alter ion flux, membrane transport, or intracellular metabolite balance may inadvertently activate this pathway. This highlights its significance in strain engineering.^108,109^

In *S. cerevisiae*, HOG signaling is initiated via two upstream branches: the Sln1 phosphorelay system and the Sho1 membrane-associated branch.^110,111^ Both pathways converge on the MAPK kinase Pbs2, which phosphorylates and activates Hog1 ^110,112^ . Once activated, Hog1 translocates to the nucleus. There, it modulates chromatin structure and induces genes required for osmoadaptation,^108,113^ such as GPD1 and GPP2, which are involved in glycerol biosynthesis. STL1, the glycerol symporter responsible for glycerol uptake, is also induced.^109,114^ Additionally, Hog1 participates in transcription-independent processes, including cell-cycle regulation and metabolic rerouting. This facilitates rapid adaptation to osmotic changes.

HOG pathway activity is highly relevant for metabolic engineering, especially in bioprocesses characterized by high sugar, high ethanol, or other osmotically challenging conditions.^115–118^ Modulating glycerol metabolism, for example, by overexpressing GPD1 or GPD2, can elevate intracellular glycerol levels. This enhances robustness in industrial fermentations.^119,120^ However, excessive glycerol production may reduce ethanol or other target chemical titers in some hosts by diverting carbon flux. This trade-off between increased robustness and product yields requires careful strain-engineering optimization to achieve favorable economic outcomes. Collectively, these observations indicate that targeted manipulation of osmoadaptive mechanisms can improve strain performance under engineered metabolic loads.

The HOG pathway is closely integrated with other stress-response networks, including the CWI and oxidative stress pathways.^121,122^ Osmotic imbalance can lead to secondary effects, such as membrane remodeling and accumulation of reactive oxygen species. Hog1 activation also influences multiple downstream stress-response modules.^123^ The principle that “membrane tweaks ripple through ROS defenses” underscores the interconnectedness of these responses. Therefore, engineering strategies that modify membrane transport, redox balance, or osmolyte flux should account for HOG signaling dynamics when evaluating potential stress burdens introduced by synthetic constructs.

### 2.6 Metabolic Stress and Energy Homeostasis: Optimizing Resource Allocation

In engineered yeast, metabolic stress often arises from increased biosynthetic and proteostatic demands due to synthetic constructs. These demands raise ATP consumption and disrupt ATP/ADP/AMP ratios. This disruption activates the Snf1/AMPK pathway, which is the primary regulator of carbon and energy status in *S. cerevisiae*.^124–126^ Activated Snf1 shifts metabolism. It represses energy-intensive anabolic processes, such as ribosome biogenesis, lipid synthesis, and amino acid biosynthesis. At the same time, it promotes ATP-generating catabolic pathways, including respiratory metabolism and the use of alternative carbon sources. These adjustments conserve ATP, stabilize the NAD^+^/NADH balance, and help maintain energetic viability during carbon limitation, overflow metabolism, or ATP depletion caused by synthetic constructs.

Synthetic burdens create competition for carbon flux among biomass formation, heterologous product synthesis, and stress-survival pathways. During energy limitation, yeast uses reserve carbohydrates, such as trehalose and glycogen, as quickly mobilized sources of carbon and ATP.^39,127–129^ Trehalose also acts as a chemical chaperone. It helps mitigate proteotoxic stress when ATP-dependent chaperone systems are impaired. This creates a link between metabolic imbalance and the HSR.^39,130^ High-flux engineered pathways may intensify metabolic stress by depleting essential precursors or changing redox cofactor availability. Both factors can contribute to ATP generation and to imbalances in NAD^+^/NADH cycling.^131,132^

The Sc2.0 minimal-genome project^133^ shows how energy- and stress-buffering networks are sensitive to genomic streamlining. The project aimed to create a chassis that was both predictable and efficient. However, systematic deletion of nonessential genes increased sensitivity to oxidative stress and reduced heterologous protein secretion.^134–136^ These results reflect the unintended loss of stress-protective modules, including chaperones and antioxidant systems. These modules buffer redox imbalance and proteotoxicity during metabolic load. Reintroducing specific chaperones and antioxidant factors restored stress tolerance and secretion capacity in engineered Sc2.0+ strains.^134,137^ These findings highlight important design principles. Keeping carbon-flux redistribution nodes, redox balance systems, and energy homeostasis networks is essential for effective metabolic engineering. Retaining these modules turns cautionary observations into actionable guidelines for building a resilient minimal yeast chassis.

In summary, the interaction among Snf1 signaling, carbon-flux redistribution (altering the flow of carbon within cells), redox balancing (maintaining stable NAD^+^/NADH ratios), and reserve-carbohydrate metabolism (managing energy storage molecules) constitutes the foundation of yeast’s adaptive response to metabolic burden. These mechanisms are critical for maintaining ATP stability and preventing resource depletion during high-burden production processes, and they inform key design strategies for engineering robust yeast chassis.^138–140^ Effective implementation of these strategies involves: 1. Fine-tuning Snf1 signaling to optimize energy allocation; 2. Modulating carbon-flux redistribution pathways to balance growth and production; 3. Enhancing redox balancing systems to stabilize NAD^+^/NADH ratios; and 4. Utilizing reserve-carbohydrate metabolism networks to strengthen energy reserves. Adoption of these targeted interventions provides a practical framework for improving the resilience and efficiency of yeast chassis in industrial applications.

### 2.7 Interconnectedness of Stress Responses: A Systems Perspective

In *S. cerevisiae*, stress responses are integrated within a complex and dynamic regulatory network termed the “stressome”.^141,142^ A comprehensive understanding of the molecular cross-talk and feedback mechanisms among distinct stress pathways is essential for developing robust, predictable engineered strains, underscoring a significant challenge in metabolic engineering. Multiple examples demonstrate these sophisticated interdependencies. Yeast stress responses involve a network of interconnected pathways characterized by key interactions and feedback loops. The HSR, regulated by the transcription factor Hsf1, is strongly activated by oxidative stress, which directly links redox balance maintenance to proteostasis. For instance, enhancing oxidative stress tolerance may be achieved by overexpressing specific chaperones that stabilize proteins during redox imbalance.^143^ The UPR is activated to resolve ER stress, and its increased demand for cytosolic chaperones often triggers the HSR, illustrating coordination between these pathways.^144,145^ Therefore, engineering strategies that upregulate UPR components may indirectly strengthen the HSR, improving overall cellular resilience. Additionally, oxidative stress complicates these interactions, as the oxidative environment required for ER protein folding generates ROS.^54^ ROS can impair cell wall integrity, which in turn activates the CWI pathway^121^ (Figure 1).

**Figure 1:**
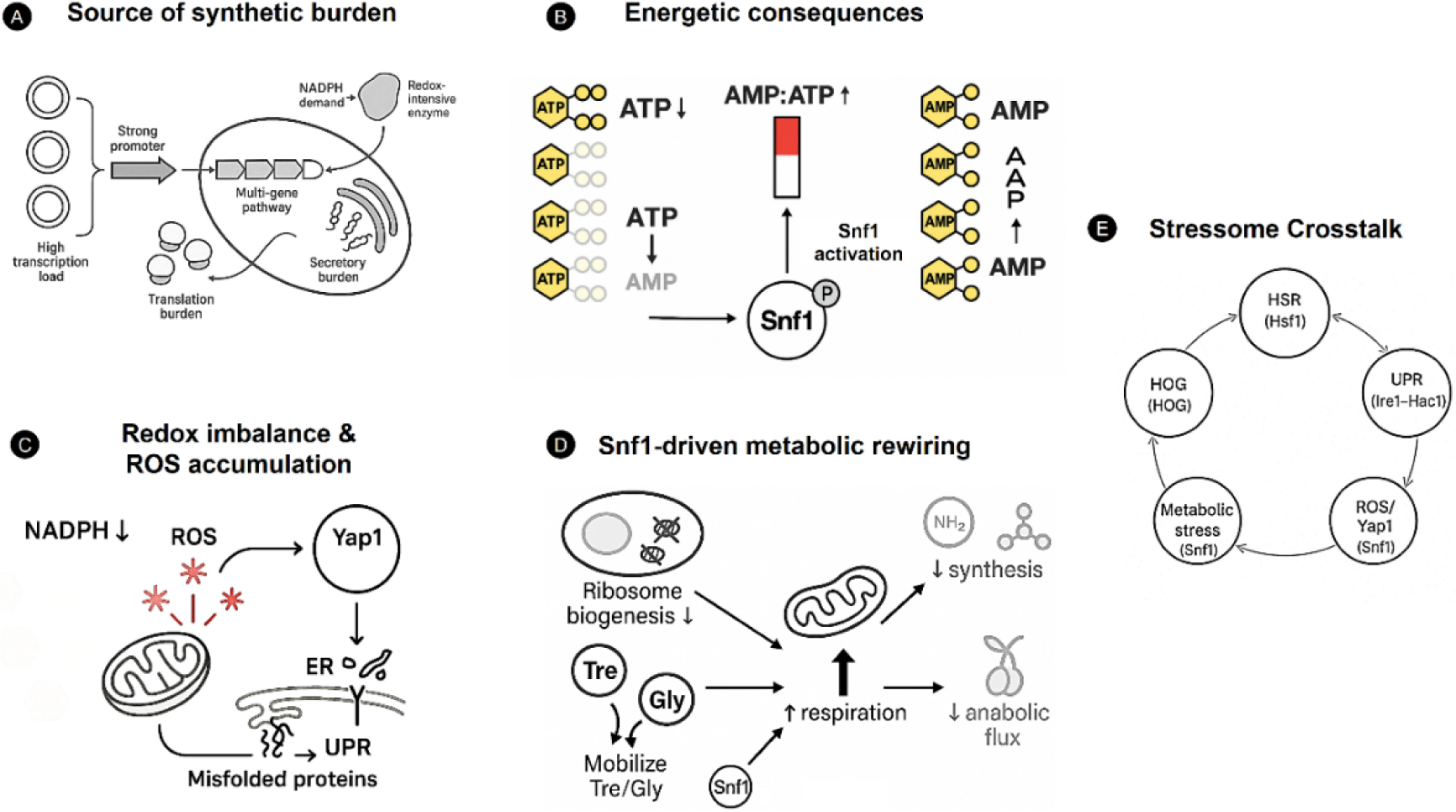
Mechanistic landscape of metabolic stress in engineered *S. cerevisiae*. The figure presents the principal cellular mechanisms by which synthetic constructs induce metabolic stress in yeast. **A** outlines the primary sources of expression burden. These include high-copy plasmids, strong promoters, multigene pathways, redox-intensive enzymes, and secretory protein overload. **B** depicts the energetic consequences of this burden. These consequences involve increased ATP consumption and elevated AMP:ATP ratios. Such changes activate Snf1 kinase and indicate intracellular energy depletion. **C** describes redox imbalance from NADPH depletion and mitochondrial ROS leakage. This imbalance triggers Yap1 activation and the propagation of oxidative, proteotoxic, and ER-stress pathways. **D** shows Snf1-mediated metabolic rewiring. Markers include reduced ribosome biogenesis, mobilization of storage carbohydrates, redirection of metabolic flux toward respiration, and widespread suppression of anabolic processes. **E** synthesizes these responses into a unified “stressome.” This includes the heat shock response, oxidative stress defenses, unfolded protein response, metabolic stress signaling, and cell-wall integrity pathways. All are coordinated through multilayered feedback interactions.

Activation of the CWI pathway modulates the HOG pathway through glycerol accumulation, linking cell wall stability to osmoregulatory responses.^146^ Similarly, under metabolic stress, the accumulation of metabolites such as trehalose affects the HSR by acting as molecular chaperones that support proteostasis. Together, these interactions within the stressome highlight the need for a holistic approach to strain development that considers the dynamic nature of these pathways.^147^

The extensive interconnectedness among stress pathways demonstrates that modifying one pathway inevitably affects others. Consequently, strain development should employ a holistic, systems-level approach rather than optimizing individual pathways independently. Adopting this strategy requires considering new design principles that emphasize pathway synergy. Identifying combinatorial interventions that effectively leverage these interconnections may enhance the robustness and efficiency of engineered strains.

## 3 Challenges and Mitigation Strategies

Although significant progress has been made, persistent challenges and research gaps remain in metabolic engineering approaches that leverage yeast stress responses. Recognizing the interconnected nature of stress pathways, this section delineates unresolved issues and proposes targeted mitigation strategies to advance the field. Each strategy is associated with explicit success metrics to facilitate rigorous evaluation of feasibility and impact.

### 3.1 Quantitative Modeling of Stress–Productivity Trade-Offs

A primary challenge in developing stress-aware strains is the lack of robust, predictive models that quantify the trade-offs between cellular stress and production yield.^9^ Synthetic burden results from a complex, nonlinear interaction among intersecting pathways and competition for limited resources. It is not a simple linear relationship.^20,148^ Extensive genetic analyses show that protein burden and nuclear export overload disrupt multiple cellular processes in *S. cerevisiae*. This underscores the multifaceted nature of synthetic burdens.^149^ Most existing models do not capture this complexity, which limits predictions of optimal expression levels, gene copy numbers, or promoter strengths for efficient pathway performance.^150^ The analogy of a conductor orchestrating a symphony helps illustrate this point. Each instrument is like a distinct pathway, such as ribosomes, chaperones, or transport vesicles. Multiscale models serve as detailed orchestral scores. They guide resource allocation more precisely than simplified approaches, which may overlook critical dynamics. Widely used modeling platforms, such as COBRA (Constraint-Based Reconstruction and Analysis) and OptRAM, provide accessible frameworks for predictive, stress-aware strain design. These platforms give researchers tools to simulate metabolic fluxes and analyze resource allocation in complex biological systems (Figure 2).

**Figure 2:**
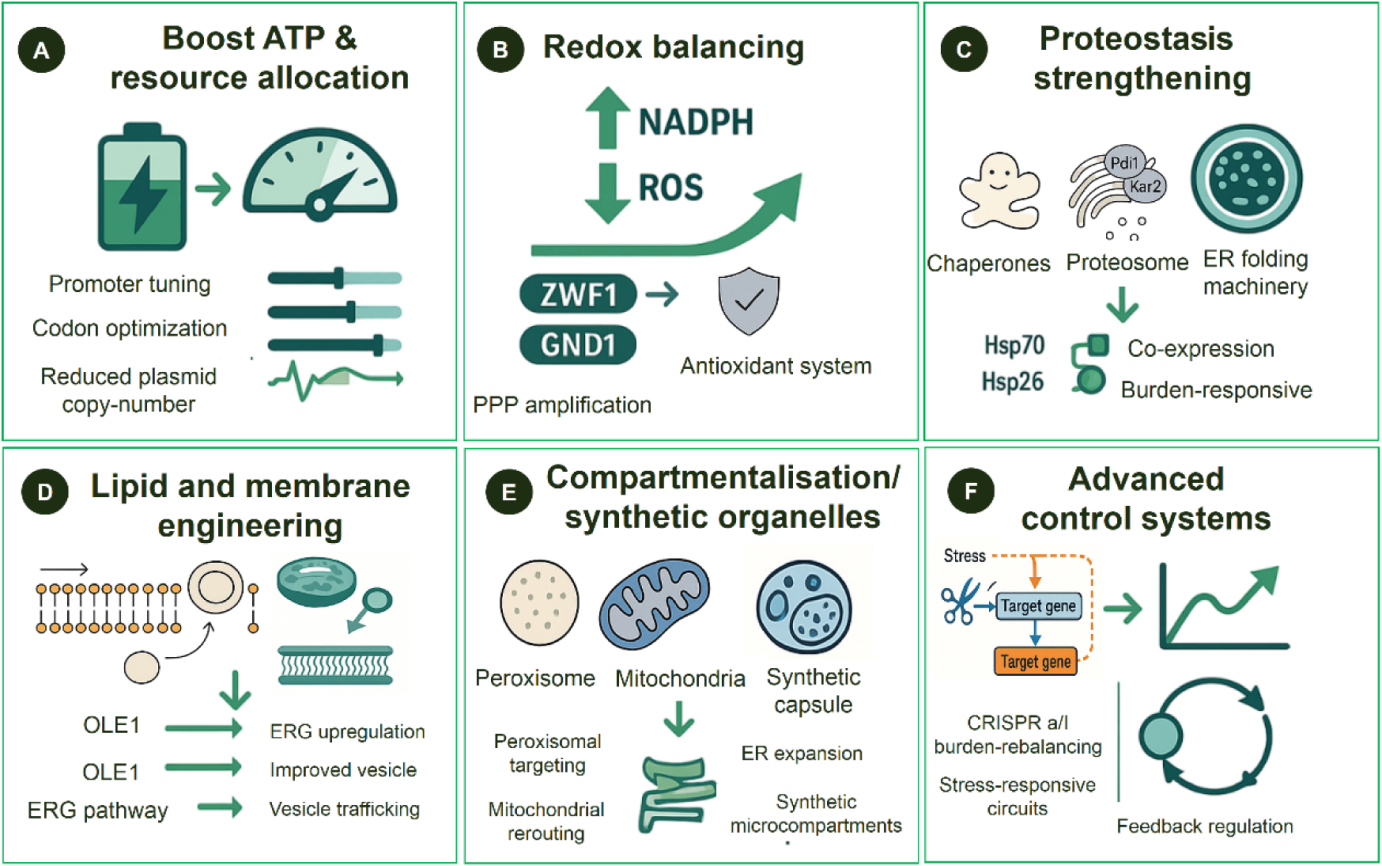
Engineering strategies to overcome metabolic stress in engineered *S. cerevisiae*. The figure summarizes key biodesign interventions that address energy, redox, and proteostasis bottlenecks associated with synthetic constructs. Section **A** details approaches to increase ATP availability and optimize resource allocation, including promoter tuning, codon optimization, reduction of plasmid copy number, and dynamic expression control. Section **B** describes redox-balancing strategies, such as amplification of pentose phosphate pathway enzymes (ZWF1, GND1), enhancement of NADPH regeneration, and reinforcement of antioxidant systems, including SOD2 and CTA1. Section **C** addresses proteostasis improvement through coexpression of chaperones (Hsp70, Hsp26), enhancement of proteasome and endoplasmic reticulum (ER) folding capacities (Kar2, Pdi1), and activation of the unfolded protein response via HAC1s. Section **D** presents lipid and membrane engineering strategies, including modulation of OLE1 and enzymes of the ergosterol pathway to improve membrane fluidity, ER function, and vesicle trafficking. Section **E** highlights compartmentalization-based interventions, such as peroxisomal and mitochondrial rerouting, ER expansion, and use of synthetic microcompartments to reduce molecular crowding and sequester toxic intermediates. Section **F** introduces advanced regulatory systems based on CRISPRa/i, stress-responsive circuits, and feedback control loops that dynamically adjust pathway expression to maintain cellular homeostasis.

Industrial production of α-amylase exemplifies the practical use of these models. This enzyme is widely used in brewing, baking, and biofuel industries. Its production is often constrained by protein folding and by ER capacity. Activation of the UPR through HAC1 overexpression has been shown to enhance secretion yields.^14,52^ Multiscale models can include these stress-aware adaptations by balancing promoter strength, gene copy number, and chaperone activity. They also consider ATP and proteostasis costs.^49,148^ Instead of relying on trial-and-error approaches, these models offer systematic pathways for developing design guidelines for energy allocation in engineered strains.

For example, upregulating a chaperone gene can reduce proteotoxic stress, but this action may divert ATP from growth and product synthesis, leading to complex, unpredictable trade-offs.^151,152^ These trade-offs reveal a research gap in multiscale, multiomics models. Such models should integrate gene expression, metabolite flux, and protein–protein interactions to predict the effects of synthetic constructs.^126,138^

Recent advances in context-dependent redesign of gene circuits emphasize the growing importance of predictive modeling. These models should account for both intracellular resource constraints and environmental variability to ensure robustness.^153^ To begin, initial models can focus on quantifiable factors such as ribosome availability, intracellular pH, and nutrient uptake rates. Addressing these factors lays a foundation for expanding to broader context dependencies. By adopting such models, engineers can move beyond empirical trial-and-error approaches and toward a predictive design cycle (Figure 3).

**Figure 3:**
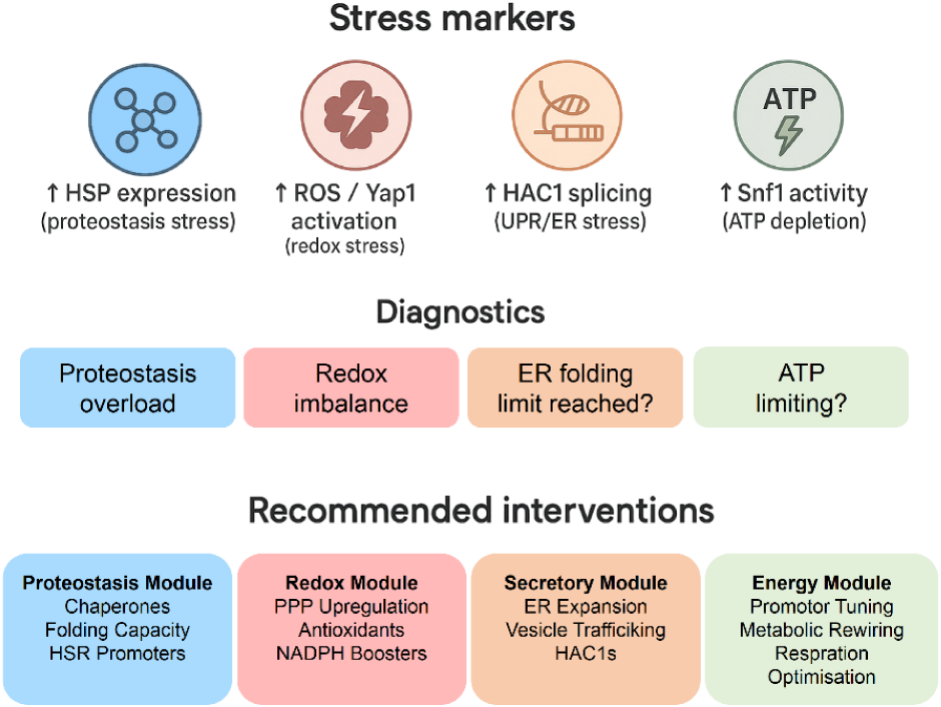
Stress-aware design framework for yeast metabolic engineering. This figure presents a diagnostic decision-tree framework linking specific stress markers to targeted intervention strategies. The top row identifies measurable indicators of cellular stress, including elevated HSP levels (proteostasis stress), increased ROS and Yap1 activation (redox stress), HAC1 mRNA splicing (ER/UPR activation), and Snf1 activation (ATP depletion). The middle row provides decision points that differentiate proteostasis overload, redox imbalance, ER folding limitation, and energy scarcity. The bottom row outlines corresponding intervention modules: a proteostasis module that enhances chaperone abundance, protein-folding capacity, and HSR-based promoter activity; a redox module that reinforces pentose phosphate pathway flux, antioxidant defenses, and NADPH regeneration; a secretory module that expands ER capacity, strengthens vesicle trafficking, and activates HAC1s; and an energy module that employs promoter tuning, metabolic rewiring, and optimization of respiratory metabolism. Together, these components form an integrated stress-aware design paradigm for guiding rational metabolic engineering in yeast.

This progression represents a transition from empirical observation to predictive engineering.^132^ Currently, strain engineering remains largely descriptive and reactive, relying on iterative trial-and-error practices.^20^ However, the envisioned path is distinctly forward-looking: to develop a predictive, engineering-based discipline in which cellular responses to synthetic burdens can be accurately modeled and designed in silico. This shift moves the field from merely understanding stress responses to actively shaping them, aligning with the central objectives of synthetic biology.^132,139,153^

## 4 Future Directions: An Integrative Approach to Stress-Resilient Strain Design

To realize the potential of *S. cerevisiae* strain design over the next decade, an integrative approach is essential, one that combines stress biology, predictive modeling, and dynamic control. The next phase of metabolic engineering in *S. cerevisiae* must move beyond descriptive analyses of stress responses to focus on predictive, integrative, and adaptive strategies. Achieving this requires integrating mechanistic insights into cellular stress biology with advanced technologies and developing yeast strains that sustain high productivity under stress imposed by synthetic constructs (Table 1).

**Table 1:** Interactions between Synthetic Constructs and Yeast Stress Responses: Key Studies and Design Guidance

Construct Type	Stress Pathway(s) Activated	Mechanistic Basis of Activation	References	Practical Engineering Design Guidance
High-copy plasmids and multigene expression cassettes	HSR; metabolic/energy stress (Snf1); secondary UPR	High transcription/translation flux overloads cytosolic chaperones (Hsp70/Hsp90) → Hsf1 activation; elevated protein synthesis and turnover increase ATP demand and activate Snf1; large secretory load or ERAD overflow can secondarily engage UPR	[^8^], [^9^], [^11^], [^20^], [^22^]	Reduce copy number or integrate into genome; use medium-strength or burden-aware promoters; implement burden-feedback controllers (e.g., burden-responsive promoters); deploy dynamic expression (induction postgrowth).
Strong constitutive promoters/high translation initiation	HSR; metabolic stress (Snf1)	Rapid translation increases nascent non-native intermediates and proteostasis demand, depleting chaperone capacity and ATP	[^7^], [^23^], [^29^]−[^30,31,32,33,34^]	Replace with tunable or stress-responsive promoters; use 5′UTR/codon-usage tuning to moderate translation rates; screen promoter variants with HSR reporters.
Heterologous secretory proteins (high-level secretion)	UPR (Ire1–Hac1); HSR (via ERAD spillover); oxidative stress (ER oxidative folding)	ER folding overload saturates Kar2/Pdi1 and ERAD—Ire1 activation and HAC1 splicing; disulfide bond formation (Ero1) generates ROS linking UPR to oxidative stress; ERAD saturation can cause cytosolic misfolded protein spillover—HSR	[^12^], [^13^], [^14^], [^24^], [^27^], [^48^], [^51^], [^52^]	Coexpress HAC1s or ER chaperones (KAR2/BiP, PDI1) transiently; improve vesicle trafficking and secretion machinery (e.g., SSO2); engineer ER membrane expansion (INO1-pathway modulation) or partition secretion via compartmentalization (peroxisomes); use inducible secretion regimes to avoid chronic UPR.
Engineered membrane proteins/membrane-targeted constructs	CWI—HOG crosstalk; oxidative stress (secondary)	Membrane crowding or altered lipid composition perturbs membrane order—Wsc/Mid sensors—Rho1–Pkc1–Slt2 (CWI) activation; membrane perturbation and lipid peroxidation from ROS couple to oxidative responses; osmotic/ionic imbalance can recruit HOG signaling	[^86^], [^100^]−[^101,102,103,104,105^], [^110^]	Prefer low-aggregation membrane variants; optimize TM domain hydrophobicity and trafficking signals; tune expression level (single copy/genomic integration); modulate lipid homeostasis (upregulate OLE1/desaturases or adjust ERG pathway genes) and assay CWI reporters during scale-up.
High-flux metabolic pathways (NAD(P)H-intensive; solvent pathways e.g., isobutanol, fatty-acids)	Oxidative stress (Yap1/Skn7); metabolic/energy stress (Snf1); HSR (via protein damage)	Pathway activity drains NADPH/NADH pools and increases electron leakage in mitochondria/peroxisomes—ROS formation; redox imbalance impairs folding and damages proteins → oxidative stress response and secondary proteostatic load; high ATP drain activates Snf1	[^15^]−[^16,17,18^], [^75^]−[^76,77,78,79,80,81,82,83,84,85^], [^79^]	Increase NADPH regeneration (overexpress ZWF1, GND1; reroute flux to PPP); coexpress antioxidant enzymes (SOD1/2, CTA1, GPX); compartmentalize reactions (peroxisomes) to isolate toxic intermediates; apply dynamic regulation to limit flux during sensitive growth phases.
Ion/osmolyte transporters or constructs altering turgor/glycerol balance	HOG (Hog1); secondary CWI activation	Altered membrane transport or osmolyte production perturbs turgor/osmotic balance—Sln1/Sho1 sensors activate Hog1 cascade; glycerol synthesis (GPD1/2) induction and transporter changes affect cell-wall coupling	[^111^]−[^112,113,114,115,116^], [^106^], [^117^]	Avoid unbalanced transporter overexpression; pretest osmotic load in process-relevant media; overexpress or tune GPD1/GPD2 or express glycerol facilitators (STL1) to stabilize turgor; monitor Hog1 reporters.
Genome minimization/streamlined chassis (Sc2.0, minimal genomes)	Increased sensitivity: oxidative stress; HSR; metabolic stress (Snf1)	Removal of nonessential “stress buffering” genes (e.g., chaperones, antioxidant systems, repair enzymes) reduces cellular capacity to absorb construct-imposed perturbations; metabolic rewiring may expose cofactor/repair deficits	[^204^], [^205^], [^207^], [^212^]−[^213,214^]	Reintroduce or retain key stress-buffer genes (e.g., HSP70 family members, SOD1); validate minimal strains with stress-marker panels; use ALE to recover missing buffering capacity and characterize adaptive alleles prior to industrial deployment.
Synthetic compartmentalization/microfactories (peroxisomes, membraneless organelles)	Primarily reduces UPR/oxidative stress and metabolic cross-talk; may create local proteostasis demands	Compartmentalization isolates toxic reactions and reduces cytosolic/ER burden; creation of new compartments can impose localized folding/transport demand and require membrane/trafficking resources.	[^170^], [^172^], [^174^]−[^175,176,177,178,179,180^]	Design import/export signals to minimize trafficking bottlenecks; size and capacity engineering of compartments (peroxisome biogenesis factors) to match pathway flux; couple compartment expression levels to local folding capacity (cotarget chaperones).

### 4.1 Stress-Aware Circuit and Pathway Design

Stress-aware circuit and pathway design requires explicitly considering how synthetic pathways disrupt cellular physiology through metabolic, proteostatic, and resource-based burdens. In engineered *S. cerevisiae*, heterologous expression costs arise not just from ATP, NADPH, and precursor depletion, but also from competition for transcriptional and translational resources. High-strength promoters and multigene overexpression draw RNA polymerase, ribosomes, and chaperones, causing resource-mediated retroactivity: circuit expression alters proteome allocation, reducing growth and efficiency.^154^ Pathway flux adds to this burden by increasing redox and cofactor demands, which trigger stress regulators such as Yap1, Msn2/4, and Snf1. Sensitivity to burden varies across pathways, so promoter strength, enzyme turnover, and flux distribution should match, not exceed, the cell’s capacity.

Dynamic regulation offers a systematic way to couple pathway output with real-time cellular states. Stress-responsive promoters, engineered with Hsf1-, Yap1-, or UPR-responsive elements, act as tunable expression valves. They reduce expression during high-burden periods and restore production once homeostasis returns.^155,156^ These closed-loop systems prevent the buildup of unfolded proteins, toxic intermediates, or redox imbalances at critical growth times. Deciding when dynamic control is better than static promoters calls for metrics that capture how control performance interacts with burden. Sensitivity shows how much promoter activity changes with stress load, often seen as the slope of the input–output curve. Latency measures the delay between stress onset and promoter actuation, such as flux-induced redox imbalance or proteotoxicity. Minimizing this delay is key to preventing irreversible growth defects. Robustness is the circuit’s ability to function well despite noisy gene expression or varied environmental conditions, common in industrial reactors.^157^ Mapping pathway burden to these metrics gives a quantitative way to pick regulatory architectures that ensure stability and productivity.

CRISPR activation and interference (CRISPRa/i) systems broaden options for stress-aware regulation. dCas9-based transcriptional modulators enable precise, reversible tuning of nodes within stress and metabolic networks. These include Hsf1 targets, Yap1-mediated oxidative stress responses, and competing branch pathways. CRISPRa/i layers can be orthogonal to native stress pathways. This reduces crosstalk and lowers the risk of unwanted regulatory loops, an important strategy for keeping stable strain performance.^158,159^ Multiplexed gRNA delivery coordinates upregulation of chaperones or detoxification enzymes and downregulation of metabolic bottlenecks. This scalable method balances proteostatic and metabolic load in real time. CRISPRa/i thus works with stress-responsive promoters, providing orthogonal, layered control within a single regulatory framework.

Using these stress-aware control strategies in genome-scale models will help rational strain development. Traditional genome-scale models describe flux distributions. Enzyme-constrained genome-scale models (ecGEMs) add proteome allocation costs. This lets us predict how synthetic circuits affect host metabolism.^126,148^ Adding kinetic properties of stress-responsive promoters and CRISPR-based controllers allows simulation of trade-offs among flux, burden, and viability. This connection links stress biology to practical engineering, enabling the design of strains that remain robust under the changing bioprocess conditions typical of industrial fermentation.

### 4.2 Rational Rewiring of Stress Pathways

Traditional engineering often relies on continuous overexpression of chaperones or antioxidant enzymes to enhance stress tolerance in yeast.^139,144^ However, this approach can cause persistent activation of regulators such as Yap1, Hsf1, or Hac1. These regulators trigger broad transcriptional changes and create a significant metabolic burden. The burden diverts RNA polymerase II and translational resources from essential housekeeping processes.^52,158,160^ These side effects harm cellular growth, disrupt redox homeostasis, and limit recombinant protein secretion. Thus, more precise and conditional control of stress responses is needed.

Dynamic and inducible regulation offers a logical alternative, enabling stress responses to activate only when specific physiological thresholds are reached. Stress-responsive promoters such as HSP26, HSP82, SSA3, and HSP12 provide native feedback by directly linking transcriptional activation to proteotoxic or thermal stress signals via Hsf1.^144,161^ Similarly, Yap1-dependent promoters respond to oxidative stress by ensuring that detoxification and redox-balancing genes are expressed only in the presence of reactive oxygen species.^162^ Together, these systems act as endogenous early warning modules that maintain basal transcriptional homeostasis and allow rapid activation of defensive pathways when needed. Mapping stress-responsive promoter activity to downstream changes in the proteome or fluxome reveals systemic adjustments in protein expression and metabolic pathways that enhance cellular resilience.

Synthetic inducible systems allow for the temporal separation of biomass accumulation from stress-mitigation programs, enabling more efficient growth and targeted stress responses. Effective replication relies on two guiding principles. First, maximizing orthogonality ensures inducible systems function independently from native cellular processes, minimizing unintended interactions. Second, minimizing basal leakiness reduces background expression in the absence of inducers, conserving resources and lowering stress. Galactose-inducible (GAL1) and copper-inducible (CUP1) promoters demonstrate tight regulation and a broad dynamic range, enabling selective activation of stress regulators without permanently altering native circuits.^164,165^ Other modalities, such as doxycycline-dependent systems^166^ and dynamic sensor-regulator circuits that connect product accumulation to transcriptional control,^167^ further illustrate these benefits. These approaches can reduce metabolic load and extend productive lifespan by preventing chronic stress signaling.

Inducible and stress-coupled networks work as transcriptional “circuit breakers.” They restrict activation of global stress programs, such as ribosomal repression, cell-cycle arrest, or metabolic rerouting—effects often seen with constitutive expression. Limiting activation to relevant conditions conserves resources, reduces crosstalk, and enables more predictable phenotypic outcomes in engineered cell factories. Still, there are potential trade-offs. These may include delayed response times or incomplete protection against stress, both of which must be carefully considered. Balancing these benefits and limitations is essential for effective stress management in engineered systems.

Emerging genome-editing tools enhance the rational redesign of stress circuits. CRISPR-dCas9 transcriptional tuning, base editing, and targeted adjustment of transcription factor binding sites allow precise changes in gene regulation without fully turning pathways on or off.^123,168–170^ These approaches provide fine control over transcription factor function, lessening chronic signal side effects and boosting responsiveness during production phases.

Computational modeling and systems-level analyses are critical for predicting promoter strength, induction thresholds, and global transcriptional load. These analyses help to find intervention points with minimal off-target effects.^139,168,169^ The predictions are tested through experimental assays, which confirm the accuracy of in-silico models. This ensures that computational insights can guide in vivo applications. Agreement between predicted outcomes and experimental data increases confidence in these models for engineering applications. Together, these tools create a rational framework. This supports engineering robust, stress-tolerant yeast strains with precise, resource-efficient regulatory behaviors.

### 4.3 Exploiting Synthetic Organelles and Compartmentalization

The compartmentalization provided by synthetic organelles offers a strategic solution for mitigating cellular stress caused by synthetic constructs. Engineered organelles, such as peroxisomes, mitochondria, and ER vesicles, enable the isolation of metabolic pathways, separating toxic intermediates and keeping ROS from the cytosol.^171,172^ This spatial segregation limits the impact of stress-sensitive reactions on essential cellular processes, thereby increasing product yields and improving strain robustness.^173,174^ For example, placing fatty-acid biosynthetic pathways in peroxisomes in *S. cerevisiae* can reduce metabolic stress from competing reactions and increase product titers by up to 700%, demonstrating the benefits of spatial segregation.^175^ Although UPR markers are reduced in strains with engineered ER morphology, there is little direct evidence linking these reductions to synthetic organelle strategies.^176^ Finally, the creation of specialized intracellular microfactories or reaction vessels further optimizes biochemical reactions, protects host cells from harmful intermediates, and increases local enzyme concentrations.^172,177^

Protein-based microcompartments and engineered peroxisomes can encapsulate entire metabolic pathways. These structures protect host cells by containing toxic intermediates. They also boost local enzyme concentrations and improve pathway flux.^177–179^ Directing synthetic pathways to peroxisomes can increase fatty-acid-derived compound production by up to 700% and reduce unwanted byproduct formation from competing enzymes.^175^ Future research should create organelle engineering design principles. These include controlling organelle size, customizing import and export, and integrating with native cellular compartments.^180,181^

### 4.4 Leveraging Adaptive Laboratory Evolution (ALE)

ALE represents a robust and complementary approach for enhancing cellular resilience under stress by enabling the emergence of beneficial mutations under defined selection pressures, without necessitating prior knowledge of underlying mechanisms.^182–186^ In yeast systems carrying synthetic constructs, ALE is particularly effective at identifying compensatory adaptations that restore essential cellular functions, including proteostasis, redox homeostasis, ion balance, and metabolic flux distribution. These processes are often challenging to predict or address comprehensively through rational design alone.^139,184,186^

Traditional engineering approaches depend on existing knowledge of gene function and regulatory networks, whereas ALE can uncover nonintuitive evolutionary solutions such as regulatory rewiring, modified transporter activity, and stress-specific metabolic reprogramming.^139,185^ The success of ALE in strain development, however, depends on the precise alignment of selection conditions with the intended engineering objective. Unstructured evolutionary pressure may result in adaptations that enhance general fitness but compromise the functionality of the engineered pathway (Table 2).

**Table 2:** Summary of Yeast Stress Responses and Engineering Strategies

Stress Pathway	Primary Stressor(s)	Key Regulator(s)	Major Cellular Outcomes	Engineering Strategies	Specific Performance Outcome(s)
HSR	Proteotoxicity from high expression, misfolding, aggregation	Hsf1; Hsp70/Hsp90 regulatory feedback	Chaperone induction (Hsp70/SSA1, Hsp26, Hsp104), proteasome activation	Chaperone coexpression (SSA1), promoter tuning, plasmid copy-number reduction	SSA1 coexpression reduced HSP26 induction, alleviated proteotoxic stress from synthetic circuit overexpression, and partially restored growth rate under high-burden conditions^7,29,32,40^
UPR	ER overload during recombinant secretion; misfolded secretory proteins	Ire1, Hac1	Upregulation of KAR2, PDI1; ER expansion; enhanced ERAD	HAC1 overexpression, ER membrane expansion (INO1), vesicle trafficking enhancement (SSO2)	Constitutively active HAC1 increased ER folding capacity and α-amylase secretion; INO1-driven ER expansion and SSO2-mediated vesicle trafficking relieved ER stress, collectively improving secretion efficiency^13,41,42,52,64–66^
ROS	ROS accumulation from high metabolic flux, NADPH depletion, redox imbalance	Yap1, Skn7	Antioxidant induction (SOD1/2, CTA1, GPX), thioredoxin system activation	PPP amplification (ZWF1, GND1), antioxidant gene overexpression (SOD2, CTA1)	ZWF1/GND1 overexpression increased NADPH regeneration; SOD2 and CTA1 reduced oxidative damage, improving viability and boosting isobutanol pathway productivity under oxidative stress^15,67,75,80–85^
CWI	Membrane/lipid imbalance; heterologous membrane protein expression; envelope stress	Wsc1–3, Mid2, Rho1–Pkc1–Slt2/Mpk1	Remodeling of β-glucan/chitin synthesis; membrane stabilization	Lipid engineering (OLE1/desaturase upregulation), ERG-pathway tuning	OLE1 upregulation increased membrane fluidity, reduced chronic cell envelope stress, and decreased persistent CWI activation during membrane-protein burdens, improving fitness and tolerance^86,94–101^
HOG	Osmotic imbalance, ethanol/osmolyte stress, turgor pressure disruption from transporter/synthetic burdens	Sln1, Sho1, Pbs2, Hog1	Glycerol synthesis (GPD1/2), osmoprotective transcription, glycerol accumulation	GPD1 overexpression, osmolyte pathway engineering	Increased intracellular glycerol improved osmotolerance and survival in high sugar and high ethanol fermentations, stabilizing metabolism under osmotic and ethanol stress^106–120,163^
Metabolic/Energetic Stress (Snf1-AMPK)	ATP depletion, flux imbalance, synthetic-pathway overload, carbon limitation	Snf1 kinase complex	Energy reallocation, reserve carbohydrate mobilization, transcriptional rewiring	Burden-aware promoter tuning; reintroduction of stress-protection genes in minimal strains	In Sc2.0-derived minimal strains, reintroducing HSP70 and SOD1 restored oxidative/proteotoxic stress resistance and recovered heterologous secretion toward wild-type levels^124–137^

To avoid selecting stress-tolerant but nonproductive variants, ALE experiments now often use counter-screening. Here, counter-screening acts as a secondary check to eliminate variants that lose or suppress the target trait. Typically, this involves transferring evolved populations to nonselective or production-like conditions, then assessing parameters such as protein expression, pathway flux, product yield, construct stability, and plasmid maintenance. For example, biosensor-assisted ALE addresses the tolerance-production trade-off by combining tolerance evolution with high-throughput screening for the target product, isolating variants that retain or improve biosynthesis.^187^ In synthetic biology, biosensors also link production phenotypes to selectable signals, supporting retention of high-producing strains after stress evolution.^188^ Mutations that promote growth by silencing or destabilizing the synthetic construct, reducing protein expression, or broadly weakening stress responses can be systematically excluded. By linking primary selection for stress tolerance and counter-selection for function, counter-screening distinguishes productive adaptations from escape mechanisms and helps retain mutations compatible with the intended application.

A rationally designed ALE workflow for synthetic-burdened yeast, therefore, involves not only continuous or serial exposure to relevant stressors but also consistent monitoring of phenotypic and molecular indicators that reflect construct-linked stress, including changes in growth rate, markers of protein misfolding, redox imbalances, metabolic bottlenecks, and expression instability. Over successive generations, adaptive mutations accumulate, and individual clones or populations exhibiting enhanced tolerance are isolated for further analysis. The primary value of ALE is subsequently revealed through postevolutionary analytical deconvolution, in which whole-genome resequencing and precise reverse-engineering strategies (e.g., CRISPR-mediated reconstruction or site-directed mutagenesis) are used to identify causative alleles and validate their functional contribution to both stress tolerance and maintenance of synthetic performance. This approach has been successfully applied in *S. cerevisiae* to uncover adaptive mechanisms conferring tolerance to industrially relevant stresses, including lignocellulosic hydrolysate inhibitors, acetic acid, acidic pH, elevated temperatures, and aromatic alcohols.^186,189–191^ The critical role of sequencing-based genotype–phenotype linkage in ALE workflows is now well-established.^186,191^ Finally, the choice of screening or evaluation method is essential, as ineffective secondary or counter screening can lead to the dominance of fitness-enhancing but nonproductive variants, thereby obscuring truly application-relevant adaptations.^192^

To harness the industrial potential of yeast strain improvements, this approach focuses on identifying adaptive mutations that enhance stress tolerance and production efficiency. Whole-genome resequencing detects mutations that have accumulated relative to the parental strain,^182,186^ while comparative genomic analysis and variant calling distinguish candidate adaptive mutations from neutral background mutations. Recurrence of mutations across independent lineages provides evidence of a selective advantage.^182,193^ This methodology offers greater precision and speed than previous yeast ALE studies, resulting in a more rapid identification and validation cycle. To establish causality, individual mutations or defined combinations are reconstructed in the ancestral genetic background using precise genome engineering methods, most commonly CRISPR-Cas-based editing.^186,194^ This workflow facilitates the construction of strains with enhanced stress tolerance, growth, construct stability, and production efficiency.^185,186,194,195^ In select cases, transcriptomic or proteomic profiling elucidates broader regulatory and metabolic changes, revealing system-level rewiring of stress responses, transport networks, or protein quality control mechanisms.^139,149^

Once validated, functionally relevant mutations may be introduced into new or optimized strain backgrounds in a targeted manner, thereby avoiding the unintended genomic alterations that often arise during prolonged evolutionary campaigns. This process forms the basis of a hybrid engineering strategy, integrating the exploratory capacity of Darwinian selection with the precision of rational genetic design.^186,194,195^ Furthermore, systems-level data generated during ALE, including global protein expression patterns and metabolic shifts, can be incorporated into predictive modeling. For example, proteome-to-metabolome mapping has revealed characteristic metabolic reprogramming signatures in *S. cerevisiae* exposed to genetic or environmental stress, providing valuable input for machine-learning-assisted strain optimization.^149^ In this way, ALE not only generates improved strains but also contributes to a deeper mechanistic understanding to guide iterative Design–Build–Test–Learn (DBTL) cycles in synthetic biology (Table 3).

**Table 3:** Challenges, Research Gaps, and Future Directions in Stress-Resilient Strain Design

Identified Challenge/Research Gap	Proposed Future Direction/Solution	Brief Rationale/Impact
Lack of predictive models for stress–productivity trade-offs	AI-guided strain design and multiomics integration	Enables in silico forecasting of burdens and trade-offs, reducing reliance on trial-and-error and guiding rational intervention strategies.
Incomplete understanding of stress pathway crosstalk and regulation	Rational rewiring of stress pathways	Targeted tuning of master regulators (Hsf1, Yap1, Hac1) allows faster and more specific stress responses, moving beyond simple overexpression of chaperones/antioxidants.
Static control of construct expression limits resilience	Stress-aware circuits and CRISPRa/i dynamic regulation	Incorporates feedback from stress signals to modulate construct expression in real time, reducing chronic burden and improving adaptability.
ALE discoveries not fully translated into precise designs	Integration of ALE with CRISPR-based precision editing	Bridges evolutionary discovery with targeted engineering, enabling efficient transfer of adaptive alleles into industrial strains.
Toxic intermediates and metabolic flux imbalances	Synthetic organelles and compartmentalization	Spatially isolates pathways to contain toxic byproducts, optimize local enzyme concentrations, and protect host homeostasis.
Genome minimization increases vulnerability to stress	Rational minimalism in chassis design	Retains or reintroduces key stress-protective modules (e.g., HSP70, SOD1) while trimming dispensable genes, balancing simplicity with robustness.

Collectively, researchers employ ALE, counter-screening, genomic analysis, and mutation reconstruction as a coherent and scalable framework for engineering yeast strains that are more stress-tolerant, genetically defined, functionally stable, and industrially relevant.

### 4.5 Artificial Intelligence (AI)-Guided Strain Design and Omics Integration

Artificial intelligence (AI) and machine learning (ML) are now part of the DBTL cycle. These tools make data-driven, mechanistically informed strain engineering possible in *S. cerevisiae*.^196,197^ In this closed-loop system, ML models act as advanced decision engines in the Design phase. They generate ranked hypotheses evaluated and refined through iterative Build–Test–Learn cycles. In contrast, traditional heuristics, such as promoter engineering or adaptive evolution, rely on incremental trial-and-error modifications. ML instead uses predictive patterns from complex, high-dimensional biological data. AI does not replace experimental reasoning. Rather, it systematically integrates diverse data sets to inform and optimize strain engineering processes.

Within this framework, “high-potential areas” are mechanistically relevant genomic and systems-level features. These features are statistically and biologically associated with stress tolerance phenotypes. Examples include stress-responsive gene clusters coregulated in response to perturbation. Other examples include regulatory hubs such as key transcription factors (e.g., HAP, MSN, YAP, HSF families) and master kinases in stress signaling. Metabolic bottlenecks under stress, such as redox regeneration, cofactor balance, and ATP availability, also qualify. Adaptive allele hotspots found in evolved populations or industrial isolates are included as well. These regions serve as actionable intervention points. Here, rational or data-driven perturbations are most likely to yield significant phenotypic improvements.

A key advantage of ML in this context is its ability to integrate multiomics data sets. These data sets include transcriptomics, proteomics, metabolomics, and, when available, fluxomics. ML uses them to identify system-level patterns tied to stress resilience. For instance, ML has mapped quantitative proteomic profiles to metabolite states in kinase knockout strains. This approach has uncovered previously unrecognized proteome–metabolome relationships linked to metabolic reprogramming and stress buffering in *S. cerevisiae*.^198^ These methods help identify regulatory hubs and pathway interdependencies that are hard to detect with reductionist analyses.

Beyond descriptive integration, ML can identify causal variants and functional alleles tied to better performance. Researchers achieve this through complementary computational and experimental strategies. Multiomics correlation and clustering analyses reveal gene or protein modules with coordinated regulation. These modules are associated with high fitness under stress. Genome-wide association-like methods applied to diverse strain panels or evolved populations can prioritize variants based on their contribution to phenotypic variance. Experimental validation of these computational predictions uses high-throughput perturbation techniques, such as CRISPRi/a or CRISPR tiling screens targeting regulatory and coding regions. Barcoded mutant libraries are also used for pooled fitness profiling under selective conditions. Such integrated pipelines go beyond correlative inference. They systematically identify causal alleles that drive adaptive phenotypes.^138,139,195,198^

Simultaneously, ML-based models can predict phenotypic outputs directly from gene expression or multiomics signatures. This facilitates in silico triage of candidate designs before costly experiments. These predictive frameworks can estimate growth rates and metabolic outputs from transcriptomic data alone. This enables rapid down-selection of engineering strategies.^199^ In metabolic engineering, ML-guided pathway optimization has increased flux through the mevalonate pathway for terpenoid production and enhanced p-coumaric acid biosynthesis. ML has also helped identify determinants of the stress response in industrial settings.^196,200,201^

Recent developments further support integrating ML with mechanistic models such as genome-scale metabolic models (GEMs). Enzyme-constrained frameworks and neural–mechanistic hybrid systems combine biochemical accuracy with pattern-recognition capabilities, resulting in improved prediction of cellular states, metabolic burdens, and intervention outcomes compared to either approach alone.^148,202^ Integrative platforms such as OptRAM expand this concept by incorporating regulatory logic alongside metabolic constraints, thereby suggesting interventions that account for gene regulation, proteome allocation, and cellular resource balance.^203^ These hybrid strategies provide a mechanistically grounded filtration layer through which ML-prioritized targets can be evaluated before experimental deployment.

Systematic prioritization becomes essential when multiple candidate adaptive alleles are identified. Hierarchical ranking of alleles can involve estimated effect size on phenotype, centrality in regulatory or metabolic networks, conservation or recurrence across independent evolution experiments, and predicted pleiotropic consequences. Epistasis testing, performed through combinatorial perturbation or factorial design experiments, is then necessary to evaluate nonadditive interactions between alleles. Multiplex genome-editing technologies enable simultaneous introduction of multiple variants, facilitating rapid exploration of synergistic, antagonistic, or buffering relationships. Combinatorial reconstruction in isogenic backgrounds and CRISPR-based allele swapping further support causal validation and refinement of multiallelic architectures. ML-guided prioritization identifies specific genomic and metabolic targets. ALE, barcoded libraries, or genome-wide screens reveal novel adaptive variants. Precision genome editing then reconstructs and combines causal alleles to generate stable, stress-resilient phenotypes. Iterative experimental validation and omics data integration complete the DBTL loop. This enables continuous refinement of predictive accuracy and engineering outcomes. The combination of AI prediction, evolutionary selection, and precise genome manipulation provides a robust framework. This framework supports the development of next-generation yeast cell factories with enhanced performance in industrially relevant stress environments.^195,204^

### 4.6 Rethinking Minimal Genome Designs: A Rational Minimalism Approach

Genome minimization, as demonstrated by the Sc2.0 and Sc3.0 projects, aims to create simpler, more predictable synthetic biology chassis. However, experiments and comparative analyses show that excessive genome reduction can remove genes needed for stress protection and metabolic stability. As a result, strains become hypersensitive to environmental and synthetic challenges.^205–207^ Removing so-called “nonessential” genes may expose hidden weaknesses, such as missing metabolite-damage control systems, detoxification pathways, and stress-resilience factors. Together, these losses can reduce fitness in production settings.^208,209^ Further, deleting stress-defense modules can make minimal strains more sensitive to the burden from synthetic gene circuits or metabolic pathways. This raises a key question: can minimal-genome strains really tolerate synthetic stress, or do they risk greater stress from losing protective functions? Rational minimalism should therefore keep or engineer core stress-defense elements. The goal is to reduce genome size without sacrificing robust cellular resilience.

A rational minimalism strategy for chassis design is recommended. Instead of maximal genome reduction, this method keeps or reintroduces stress-protective modules, such as chaperones and antioxidant defenses, including HSP70 and SOD1. Elements that do not help robustness or predictability should be removed. Choose genes using essentiality screens and omics data to find those key to stability and function under stress. Put this strategy into practice through a DBTL cycle. Start by designing a minimal genome with essential genes, then build it with synthetic biology tools. Test its performance under real-world conditions. Refine the gene selection after reviewing the results.

To put this approach into action, use a retention scorecard. Assign each gene a score from 1 to 5 based on its stress-protection value compared to its genomic size cost. This method encourages systematic evaluation and speeds up decisions. It also avoids the need for large tables. Using a scorecard helps clarify choices about gene retention by visualizing each gene’s contribution relative to its space requirements.

Three lines of evidence support this approach. First, targeted genome trimming and refunctionalization can create compact, robust genomes.^206^ Second, metabolite-damage and repair pathways are often needed for stable minimal systems and must be part of the design.^208^ Third, evolutionary and systems analyses show that synthetic lethality and epistatic interactions limit how much you can reduce the genome without losing robustness in real conditions.^210,211^

Several technologies and workflows help enable rational minimalism. High-throughput genome-editing and plasmid-design systems enable precise, marker-free changes, reducing the time to develop and test new chassis from months to days. This highlights their powerful impact.^212^ Model-guided design uses genome-scale metabolic and regulatory models, along with experimental data, to determine which genes to keep, remove, or add to enhance resilience.^203,213^ ALE and synthetic evolution help find compensatory adaptations and hidden fitness costs. These can then be engineered into the chassis.^195,214^ Careful studies of metabolite damage, damage-control systems, and minimal-cell evolution show which gene categories are essential to keep or reintroduce.^208,215^

In summary, the best way to develop an industrial chassis is to combine genome minimization with targeted refunctionalization. This approach relies on mechanistic understanding, predictive models, automated editing, and evolutionary analysis. Rational minimalism balances predictability and simplicity, while ensuring robustness under high-flux and industrial stress.

## 5 Conclusions

Engineering *S. cerevisiae* for synthetic biology requires balancing the function of synthetic constructs with the host’s stress resilience. This review demonstrates that various synthetic constructs, including high-level secretory proteins and complex regulatory circuits, induce multiple interconnected stress responses (Figure 4). These include the HSR, UPR, oxidative stress pathways, CWI, and HOG signaling.^106,107^ While these adaptive mechanisms preserve cell integrity, they frequently reduce productivity. This underscores the inherent trade-off between cellular defense mechanisms and industrial performance.^131^

**Figure 4:**
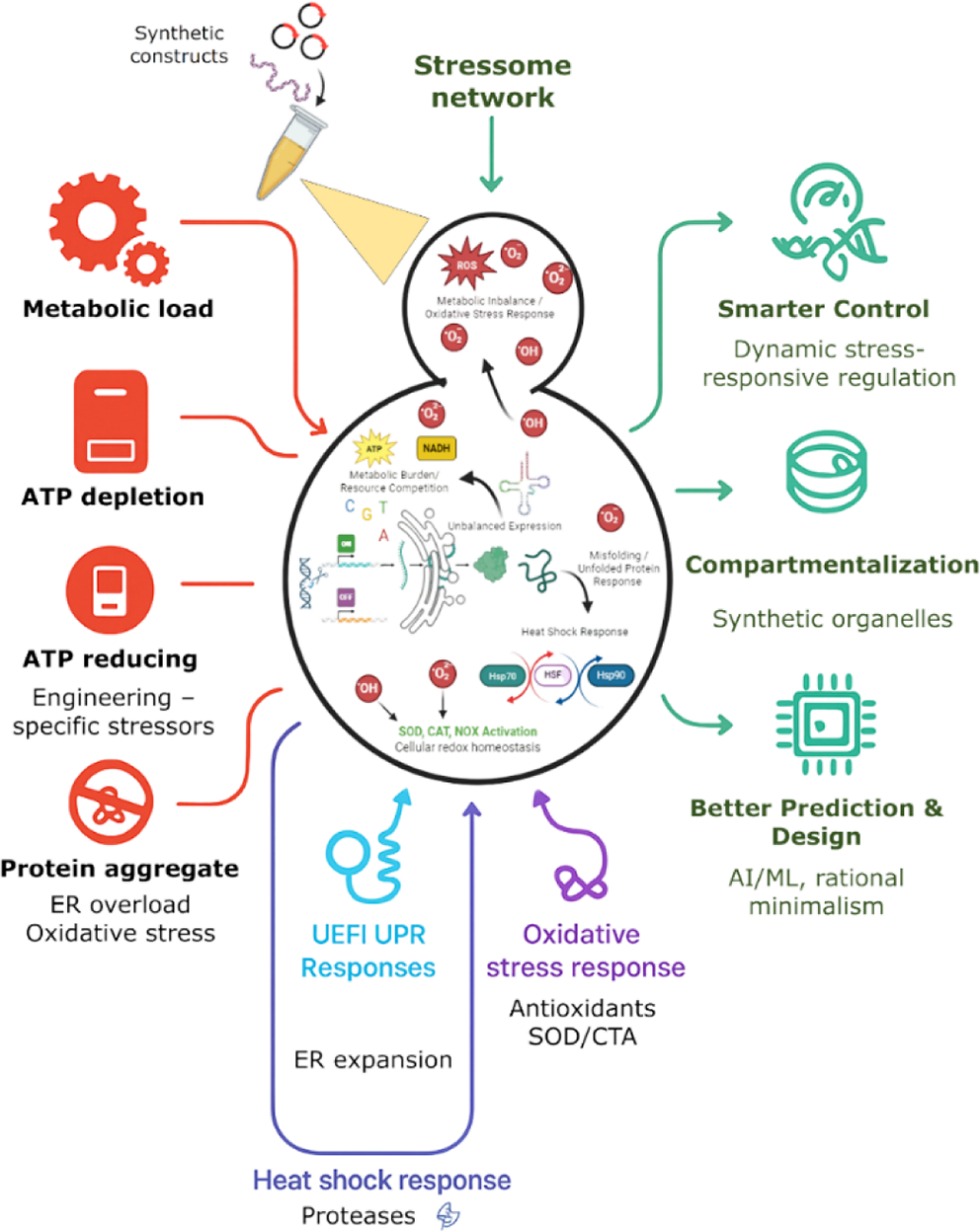
Stress responses in *S. cerevisiae*induced by synthetic constructs and their mitigation strategies. Synthetic constructs, such as plasmids, gene circuits, and engineered pathways, burden yeast cells. These burdens cause metabolic stress from resource diversion, ATP depletion, and activation of Snf1/AMPK. Proteostasis stress encompasses protein misfolding, aggregation, and the activation of the HSR and UPR. Oxidative stress results from excess ROS and redox imbalance, which activates Yap1 and Skn7 signaling. Cell wall and membrane stress arise when membrane changes trigger the CWI pathway. Osmotic stress, from ion and turgor disruptions, activates the HOG pathway. These stress pathways are interconnected, forming the “stressome” network. This maintains homeostasis but diverts resources from growth. Engineering strategies, such as chaperone coexpression, redox balancing, lipid remodeling, optimizing glycerol pathways, and stress-aware circuit design, can reduce these burdens and improve yeast robustness in synthetic biology.

Recent research supports a stress-aware design philosophy. This approach uses strategies such as promoter tuning, chaperone coexpression, dynamic regulation, and metabolic rewiring. These methods help mitigate construct-induced stress while sustaining high productivity.^127,147,154^ For example, coexpression of Hsp70 can restore yeast growth and protein secretion under stress. Trehalose accumulation acts as a chemical chaperone. This links metabolic adaptation directly to proteostasis.^127,216^ These interventions require careful optimization to prevent regulatory complications or increased metabolic burden. Case studies show that integrating mechanistic understanding with both rational and evolutionary engineering can improve the performance and stability of engineered strains.^183^

Emerging technologies offer novel solutions to existing challenges. Synthetic organelles and compartmentalization sequester stress-prone reactions.^171,173,178^ ALE with CRISPR-based genome editing enables precise stress-response tuning.^149,153,194^ Integrating AI models with multiomics profiling identifies stress hotspots early, reducing trial-and-error in strain development and improving efficiency.^148,196,200^ For example, an AI-predicted mutation can be quickly validated in a single ALE round, demonstrating the synergy between computational insights and evolutionary adaptation. Rational genome minimalism, retaining key genes such as HSP70 and SOD1, preserves stress resilience while maintaining minimal genome simplicity and predictability.^205,206^

In conclusion, making yeast a robust chassis for industrial use requires a comprehensive, integrated strategy that blends stress biology and advanced synthetic methods. By overcoming challenges such as pathway crosstalk and the trade-offs between stress tolerance and productivity, the field can develop yeast strains that are simultaneously resilient and highly productive.^107,139^ Establishing a “stressome benchmark consortium” to create shared data sets and standard assays will rapidly accelerate progress toward stress-resilient yeast. These collaborative benchmarks will set the stage for synthetic biology to fully realize yeast’s potential as a sustainable, versatile platform for producing fuels, chemicals, and therapeutics, driving impactful industrial advancements.
